# What do future public health doctors know about the One Health concept in Portugal?

**DOI:** 10.1093/eurpub/ckac131.322

**Published:** 2022-10-25

**Authors:** AM Alho, R Vasconcelos, B Gomes, AB Nunes

**Affiliations:** 1 ACES Lisboa Norte, Portuguese Association Public Health Doctors, Lisbon, Portugal; 2 ACES Oeste Norte, Portuguese Association Public Health Doctors, Caldas da Rainha, Portugal; 3 ACES Entre Douro Vouga I, Portuguese Association Public Health Doctors, Santa Maria da Feira, Portugal; 4 Portuguese Association Public Health Doctors, Lisbon, Portugal

## Abstract

**Background:**

The One Health (OH) approach brings together a transdisciplinary collaboration between human, animal, and environmental health, to tackle emerging zoonotic diseases, antimicrobial resistance, and food safety. Therefore, incorporating of OH principles in the education of health care providers is fundamental.

**Methods:**

To assess OH knowledge, attitudes and practices (KAP), an anonymous, multiple-choice, online self-administered survey was sent to 1st year Portuguese Public Health Medical Residents (PPHMR), during an online congress targeted to them. A descriptive analysis was performed.

**Results:**

A 50.0% response rate was obtained out of the 42 PPHMR attendees. Only 33.3% were familiar with OH concept; 57,1% had heard of it but were not aware of its meaning, and 9.5% had never heard of it. Concerningly, 9,5% believed zoonosis were diseases transmitted between animals and 42.9% considered that “antimicrobial resistance” is applied to antibiotics only. Regarding major zoonosis, etiologic agents were not recognized for Cryptosporidiosis (47.6%), Echinococcosis (42.9%), Toxoplasmosis and Leptospirosis (38.1%), Dermatophytosis (33.3%), Rabies (28.6%), Borreliosis/Brucellosis (23.8%). Half (52.4%) were unaware of the transmission route of Brucellosis/Dermatophytosis, followed by Leptospirosis (38.1%), Toxoplasmosis (28.6%) and Borreliosis/Rabies (23.8%). Remarkably, all participants showed willingness to be informed on OH issues and agreed that prevention and speed of intervention would be higher with greater collaboration between health technicians. About education towards OH throughout their medical curricula, 61.9% classified it as low, 23.8% as absent, 14.3% as sufficient and none classified it as adequate or very adequate.

**Conclusions:**

This is the first study assessing KAP regarding the OH concept among PPHMR. Results highlight the need to bring OH to the Portuguese medical schools’ agenda to better prepare the next generation of PPHMR to the emerging health crisis.

**Key messages:**

• Despite the interest shown by 1st year Portuguese Public Health Medical Residents concerning One Health, a general lack of knowledge on the topic was found.

• The majority qualified as insufficient their training on this subject, highlighting the need for medical schools to improve education and raise awareness regarding this transdisciplinary approach.

